# Antioxidant and antibacterial activities of the rhizome extract of *Curcuma zedoaria* extracted using some organic solvents

**DOI:** 10.5455/javar.2023.j687

**Published:** 2023-09-24

**Authors:** Agus Budiansyah, Ucop Haroen, Syafwan Syafwan, Kiki Kurniawan

**Affiliations:** 1 Faculty of Animal Husbandry, Jambi University, Jambi, Indonesia; 2 Center for Vaccine and Drugs Development, National Research and Innovation Agency, Kabupaten Bogor, Indonesia

**Keywords:** Antioxidant, antibacterial, rhizome extract, organic solvents

## Abstract

**Objective:**

This research aims to identify the effect of various organic solvents such as n-hexane, ethyl acetate (EtOAc), and methanol (MeOH) on the antioxidant and antibacterial activities of *Curcuma zedoaria* extract, against three Gram-positive bacteria, namely* Staphylococcus aureus, Bacillus subtilis, *and *Streptococcus pneumoniae*, and three Gram-negative bacteria, namely *Escherichia coli, Salmonella typhi, *and *Pseudomonas aeruginosa*

**Materials and Methods:**

As much as 1 kg of white turmeric rhizome (*C. zedoaria*) was extracted two times for 24 h using 3 l of MeOH before evaporating. The extract was then fractionated using n-hexane six times per 2 h, with each volume of 500 ml, and continued with the EtOAc fractionation. The MeOH fraction was added to 300 ml of water before adding 400 ml of EtOAc. Once the fractionation process was complete, all fractions were concentrated using a rotary evaporator.

**Results:**

The *C. zedoria* extract fractioned using MeOH produces alkaloids, phenolics, flavonoids, saponins, and coumarin compounds. The fractionation with EtOAc also produces alkaloids, phenolics, flavonoids, saponins, coumarin compounds, and triterpenoids. Meanwhile, fractionation with n-hexane only produces alkaloids and triterpenoid compounds. EtOAc and MeOH fractions had good activity in reducing free radicals produced by 2,2-diphenyl-1-picrylhydrazyl (DPPH), with an average IC_50 _value of 153.49 ± 2.66 and 185.77 ± 3.91 ppm, respectively. In contrast, the n-hexane fraction has weak antioxidant activity with an IC_50 _value of 837.92 ± 5.32 ppm. The n-hexane fraction has better activity compared to MeOH and EtOAc. The lowest concentration required was 2,500 ppm for all types of bacteria.

**Conclusion:**

*Curcuma zedoaria* extract produces alkaloids, phenolics, flavonoids, saponins, coumarins, and triterpenoids when fractionated with MeOH or EtOAc. Only alkaloids and triterpenoids are produced using n-hexane. EtOAc and MeOH fractions have good activity in reducing free radicals generated by DPPH, with an average IC_50 _value of 153.49 ± 2.66 and 185.77 ± 3.91 ppm, respectively. However, n-hexane has weak antioxidant activity, with an average IC_50 _value of 837.92 ± 5.32 ppm. All fractions have moderate antibacterial activity, but the extract of n-hexane from *C. zedoary* has better antibacterial activity compared to MeOH and EtOAc. The lowest concentration required is 2,500 ppm for all types of bacteria.

## Introduction

The use of synthetic feed additives to increase livestock production, especially poultry, has been frequently used. Synthetic feed additives are usually antibacterial, such as antibiotics and chemotherapeutics [1,2]. Apart from their function in preventing diseases, synthetic feed additives are also used to promote growth. However, the use of synthetic feed additives was banned in 2003, according to European Union No. 1831/2003. This rule was intended to prohibit the use of any antibiotics or chemotherapeutics as growth promoters because they have a negative impact on human consumption of livestock products and lead to the development of antibacterial resistance in those individuals [3]. To solve the negative impact of these antibiotics and chemotherapeutics, herbal feed ingredients or medicinal plants used as feed additives can be beneficial. The whole parts of medicinal plants, including the leaves, roots, flowers, and fruit, are commonly used to cure various diseases [4,5]. The components of active compounds in all parts of the plant are chemical compounds that have therapeutical, positive effects. The components of active plant compounds are secondary metabolites, especially glucosides, alkaloids, phenolics, terpenoids, and essential oils [6]. These active components are easy to absorb in the intestines, easily metabolized with glucocorticoids, and quickly excreted in the urine [7]. Because secondary metabolites from herbal plants are easily absorbed and quickly excreted, they are stored less in the body. Hence, there is a low risk of accumulation in body tissues, and thus the components of active compounds in herbal plants are more efficient in utilizing food substances that will increase the growth of livestock [7]. Hernández et al. [8] stated that plant extracts are proven to contain molecules that effectively stimulate digestive enzymes, resulting in a significant increase in appetite and growth.

*Curcuma zedoaria*, an herbal plant, belongs to the Zingiberaceae family and is a member of the genus *Curcuma*. The tuber of *C. zedoaria *(also known as white turmeric) is an herbal medicine and the most widely used spice in Asia, particularly in Indonesia. *Curcuma zedoaria *has various types of primary and secondary metabolites. The main components of the plant are starch, curcumin, essential oil, and gum Arabic. The rhizome of *C. zedoaria* was found to have more than 10 sesquiterpenes, including curcumin, ethyl p-methoxycinnamate, β-turmerone, β-Endemol, zingiberene, dihydro curcumin, furanodiene, α-phellandrene, 1,8-cineole, β-elements, and germacrone [9]. Setyani et al. [10] discovered that the crude extract of *C. zedoaria *rhizome contains compounds of alkaloids, saponins, terpenoids, flavonoids, and tannins depending on the polarity of the organic solvent used in partitioning or fractionating its crude extract. Almost all species of *C. zedoaria *contain active compounds that have antioxidant activities and pharmacological effects, are prospective for clinical use in the future [11], and have antibacterial activity [12].

At doses of 100, 250, 500, and 1000 mg/ml, the rhizome extract of *C. zedoaria *effectively inhibits the growth of *Streptococcus mutants*, *Enterococcus faecalis, Staphylococcus aureus, *and *Candida albicans* and could reduce as much as 99.99% of the population of microbes [12]. Another beneficial effect of this white turmeric is that it is not only used for the maintenance of physical health but is also advantageous for curing various diseases such as cancer or tumors [13,14]. Dosoky and Setzer [15] affirm that the extract of *C. zedoaria* rhizome shows anticancer characteristics, antiinflammatory, analgesic, antiallergic, antiparasitic against *Entamoeba histolytica*, antibacterial, and antifungal activity. Not only has this plant been identified as a medicinal plant, but it is also used as an organic feed additive for livestock, such as fish, with the result of increasing the immune response and is recommended to be given at 0.5 gm/kg [16]. *Curcuma zedoaria *has high antioxidant levels. In poultry, the use of its extract in combination with turmeric and garlic extract (*Allium sativum*) as additional feed additives could increase body weight and accelerate the recovery process of chickens infected with chronic respiratory disease [17].

In previous studies, the extraction of phytochemicals from the *C. zedoaria *rhizome was conducted merely using ethanol or methanol (MeOH) [14,18]. Ethanol or MeOH solvents are polar solvents that can only extract polar compounds. But the *C. zedoaria *rhizome contains not only compounds that are polar, but there are also compounds that are semipolar. Semipolar compounds can only be extracted with semipolar solvents such as ethyl acetate (EtOAc). Meanwhile, nonpolar compounds can only be extracted with nonpolar solvents, namely n-hexane. Therefore, it is necessary to use several solvents that have the ability to extract these three compounds, namely polar, semipolar, and nonpolar compounds [19]. An extraction using other solvents, such as EtOAc and n-hexane, has not been widely reported. Previous antibacterial activity tests used only one or two bacteria, consisting of Gram-positive or Gram-negative bacteria, which were carried out using discs. However, the number of bacteria that attack poultry can be more than two types of bacteria. Therefore, testing with more bacteria is needed. A test conducted using sixty bacteria at once, which consists of Gram-positive and Gram-negative bacteria, has never been done. The test in this study was carried out thoroughly using discs, minimum inhibitory concentration (MIC), and minimum bactericidal concentration (MBC) so that a thorough conclusion about the minimum concentration of *C. zedoaria *required as an antibacterial can be drawn. The purpose of this study was to scrutinize the influence of organic solvents such as n-hexane, EtOAc, and MeOH on antioxidant and antibacterial activities of *C. zedoria* extract against three Gram-positive bacteria (*S. aureus, Bacillus subtilis, *and* S. pneumoniae*) and three Gram-negative bacteria (*Escherichia coli, Salmonella typhi, *and* Pseudomonas aeruginosa*), which have the potential to be used as feed additives in poultry.

## Materials and Methods

### Ethical statement

The research was carried out at the Research Center for Vaccine and Drug Development, National Research and Innovation Agency (known as BRIN), Jalan Raya Jakarta-Bogor Km 46, Cibinong, Bogor, Indonesia, from October 2021 to March 2022. A total of 1 kg of rhizome of *C. zedoaria *that had been air-dried was gradually macerated using n-hexane, EtOAc, and MeOH. Phytochemical profile testing of rhizome *C. zedoaria *samples was conducted at the Natural Organic Chemistry Laboratory of the Indonesia Institute of Science, certified by the National Accreditation Committee [No. LP-767-IDN, Tahun 2017].

### Materials used in this study

Tools utilized in this research were an analytical balance, 500 ml beaker glass, 250 ml beaker glass, a set of distillation apparatus, vials, funnels, a set of column chromatography, Erlenmeyer-300 ml, a UV lamp with 254 and 365 nm (UV-02), tweezers, thin-layer chromatography, Petri dishes, inoculation needles, a spirit burner, 12 × 8 microplate, and spatula. Furthermore, this research also used a porcelain dish, pestles, a stirring rod, a measuring cup of 5 ml, a measuring cup of 500 ml, a chromatography development vessel, a set of rotatory evaporators, a dropping pipette, a capillary pipette, an oven, an incubator, a micropipette (100–1,000 m), and a set of UV-vis spectrophotometers (Shimadzu, 1700).

Furthermore, the materials used in this study were white turmeric (*C. zedoaria*) powder, n-hexane, EtOAc, MeOH, “Whatman” filter papers, H_2_SO_4_ 2N, NaOH, chloroform, 2,2-diphenyl-1-picrylhydrazyl (DPPH), vitamin C as a positive control, Gram-positive bacteria (*S. aureus, B. subtilis, *and* S. pneumoniae*) and Gram-negative bacteria (*E. coli, S. typhi, *and* P. aeruginosa*)*, *HCl_2_N, Dragendorff’s reagent, magnesium powder, dichloromethane, 10% of sulfuric acid, Nutrient Agar, aluminum foil, and tetracycline as positive controls.

### Maceration and fractionation of MeOH extracts of C. zedoaria rhizome

A total of 1 kg of *C. zeodoria* rhizome was extracted twice with 3 l of MeOH for 24 h each time and then evaporated. As much as 75.2 gm of MeOH extract was obtained from the process. Hereafter, the extract was fractionated with n-hexane six times per 2 h with 500 ml of n-hexane for each repetition. Once it was completed, 300 ml of water was added to the MeOH fraction to increase its polarity, after which 400 ml of EtOAc was added in a 2-h experiment. The process was repeated ten times [20]. When the fractionation process was finished, all fractions were concentrated using a rotary evaporator to obtain 43 gm of MeOH fractions, 13.4 gm of an EtOAc fraction, and 13.4 gm of n-hexane.

### Phytochemical screening

Approximately a teaspoon of MeOH, EtOAc, and n-hexane fractions of *C. zedoaria* rhizome was fed into a test tube and added to 3 ml of MeOH to be homogenized [21]. This phytochemical screening aims to identify the amount of secondary metabolite contained in each fraction. Some components of secondary metabolites in the rhizome of *C. zedoaria* are vital for the antibacterial and antioxidant activities that would be done as they provide information about the nutrition of animal feed.

### Screening of anti-bacterial activity

The study conducted a diffusion test (dilution) to determine MIC and MBC test methods for screening antibacterial activity. The diffusion test allows us to predict the concentration of antibacterial agents tested in the nutrient agar [22]. The agar dilution method was used to quantitatively gauge the *in vitro* antibacterial activity against bacteria and fungi.

### Media creation process

As much as 10 gm of nutrient agar powder is fed into the Erlenmeyer 1 l after which is then added to 500 ml of distilled water. It was stirred and heated on a hot plate, then sterilized and poured into a petri dish [23].

### Bacterial strains

Various strains of pathogenic bacteria were utilized to test the antibacterial activity of the active fractions of *C. zedoaria* rhizome. The Gram-positive bacteria strains used were *S. aureus* (FNCC-0027) and *B. subtilis* (FNCC-0035), while the Gram-negative bacteria were *E. coli* (FNCC-0145) and *P. aeruginosa* (ATCC 2467). These two types of Gram-positive and Gram-negative bacteria were obtained from Gadjah Mada University. Other bacterial strains used were *S. pneumoniae* (ATCC70184) (Gram-positive) and the Gram-negative *S. typhi* (DSM 21028), which were obtained from the Microbiology Laboratory of the Chemical Research Centre, National Research and Innovation Agency.

### Inoculum standardization preparation

A turbidity standard of 0.5 McFarland was employed to prepare the inoculum for standardization in antibacterial susceptibility. Bacteria were cultured on nutrient agar and incubated at 37ºC for 18 to 24 h. The pure colonies on the media were taken and diluted in physiological saline solution, and the turbidity was adjusted to 0.5% McFarland standardization, which is equivalent to 1 × 10^8^ CFU/ml [24].

### Antibacterial activity test by disc diffusion method

The antibacterial activity test using the disc diffusion method is an initial screening carried out to verify the potential of antibacterial activity in a fraction of samples. It used paper discs that acted as a place to house antibacterial compounds. Sterile filter papers of 6 mm were initially prepared and impregnated with 100 µl of the MeOH fraction, the EtOAc fraction, 10,000 ppm of n-hexane, 200 ppm of tetracycline that acts as a positive control, and MeOH as the negative control. Hereafter, the impregnated sterile papers were placed on the surface of nutrient agar that had previously been inoculated using a bacterial test and incubated at 37ºC for 18 to 24 h. In the last stage, the antibacterial activity was measured by placing filter papers with antibacterial compounds on top of agar media and evaluating the diameter of the inhibition zone around the growth of bacteria [25].

### MIC assay

In this test, the dilution of samples was completed using Mueller-Hinton broth to reach a concentration range of 750–100.000 ppm on microplates. The bacterial suspension was diluted in a 0.85% physiological saline solution and adjusted to produce turbidity that is comparable to 0.5 McFarland, which is equivalent to 1 × 10^8^ CFU/ml. The results were observed visually by comparing the suspensions. Subsequently, a serial dilution of samples was prepared, and a standard inoculum of bacterial strains was added to each sample concentration. The microplate was then incubated at 37ºC for 18 to 24 h. The lowest concentration in the samples that suppresses the invisible growth of bacteria is perceived as the MIC [24].

### MBC assay

In this process, the MIC of samples was plated on nutrient agar. As much as 100 l of the concentration was taken, subcultured onto the agar plate, and incubated at 37ºC for 18 to 24 h. The MBC was recognized based on the lowest concentration that did not show any bacterial growth on the newly inoculated plate [26].

### Preparation of vitamin C standard solution

Vitamin C was used as a positive control. 10 mg of vitamin C was dissolved in 2 ml of ethanol and added to distilled water to make a 100 ml solution. The solution was then gradually diluted to 50, 25, 15, and 5 ppm using a volumetric flask [27].

### DPPH free-radical scavenging activity

The DPPH free-radical scavenging test was conducted by modifying the assay protocols. The test principle was characterized by a color change from purple to yellow due to the antioxidant in the samples. The extract solution with various concentrations (10, 50, 100, and 200 µg/ml) in 2 ml MeOH was added with 0.5 ml DPPH (1 mM in MeOH). The mixture was evenly stirred and left for 30 min to react with DPPH. Absorbance was read at 517 nm and the percentage of inhibition was calculated by determining the difference in absorption between the blank solution and samples [28].


$$\mathrm{DPPH}\;\mathrm{Free}-\mathrm{radical}\;\mathrm{Scavenging}\;\mathrm{Activity}\;(\mathrm{\%)}=1-\left\{\frac{\mathrm{the}\;\mathrm{absorption}\;\mathrm{of}\;\mathrm{control}–\mathrm{the}\;\mathrm{absorption}\;\mathrm{of}\;\mathrm{sample}}{\mathrm{the}\;\mathrm{absorption}\;\mathrm{of}\;\mathrm{control}}\right\}$$

## Results and Discussion

### Phytochemical screening

*Curcuma zeodoria* rhizome powder contains various important secondary metabolites, as revealed by the phytochemical screening of its MeOH, EtOAc, and n-hexane fractions. These metabolites, including flavonoids, phenolics, and triterpenoids, indicate that the extracts have significant biological activity in terms of their antioxidant and antibacterial properties. For a comprehensive breakdown of the findings, please refer to Table 1

The test results on the extract of *C. zedoaria* designate that the extract fractionated using MeOH contains alkaloids, phenolics, flavonoids, saponins, and coumarin compounds, but not triterpenoids and steroids. When it was fractionated using EtOAc, the extract contained alkaloids, phenolics, flavonoids, triterpenoids, saponins, and coumarin compounds, yet it did not have steroids. The fractionation completed using n-hexane, however, results in only alkaloids and triterpenoids (Table 1

A different result by Setyani et al. [10] reported that the crude extract of *C. zedoaria* rhizome consists of such compounds as alkaloids, saponins, terpenoids, flavonoids, and tannins. The crude extract of three organic solvents with different polarities yields different compounds. The fractionation done using n-hexane produces terpenoids and flavonoids, while the one using EtOAc contains alkaloids, terpenoids, and flavonoids. Furthermore, the MeOH fractionation results in saponins, flavonoids, and tannins. Another study by Himaja et al. [31] reveals that the fractionation of n-hexane yields flavonoids, while the one using petroleum ether yields terpenoids and phytosterols. Moreover, the EtOAc fraction comprises phenolics and tannins, and the fractionation done using ethanol produces more compounds such as alkaloids, flavonoids, saponins, phytosterols, carbohydrates, and glycosides. In addition, the fractionation with chloroform generates compounds containing terpenoids and alkaloids. Lastly, in their study, it was also discovered that the fractionation with water produces alkaloids, phenolics, tannins, carbohydrates, and glycosides. The variation in plant secondary metabolites or phytochemical compound composition is due to factors such as solvent concentration, plant age, growth location, and soil fertility [19].

**Table 1.** The result of phytochemical screening of MeOH, EtOAc, and n-hexane.

No	Parameters	Observation	MeOH fraction	EtOAc fraction	n-hexane fraction
1	Alkaloids	Orange precipitate [29]	Orange precipitate (√)	Orange precipitate (√)	Orange precipitate (√)
2	Phenolics	Purple [29]	Purple solution (√)	Purple solution (√)	No purple solution (-)
3	Flavonoids	Orange-red colored solution [29]	Orange-red colored solution (√)	Orange-red colored solution (√)	No orange-red colored solution (-)
4	Triterpenoids	Red-purple solution	No red-purple solution (-)	Red-purple solution (√)	Red-purple solution (√)
5	Steroids	Green colored solution [29]	No green-colored solution (-)	No green-colored solution (-)	No green-colored solution (-)
6	Saponins	The foam does not disappear after the addition of concentrated HCl [29]	Foam is formed after the addition of concentrated HCl (√)	Foam is formed after the addition of concentrated HCl (√)	Foam is not formed after the addition of concentrated HCl (-)
7	Coumarins	The presence of blue-green fluorescence under the 365 nm UV lamp after it is sprayed with 2% NaOH [30]	There was blue fluorescence after spraying with 2% NaOH (√)	There was yellow-green fluorescence after spraying with 2% NaOH (√)	There was not any fluorescence after spraying with 2% NaOH (-)

The phytochemical screening was conducted at the laboratory of Natural Chemicals, Chemical Research Centre, National Research, and Innovation Agency, in 2022.

A total of 75.2 gm (7.52%) of the MeOH -concentrated extract was acquired by macerating 1 kg of *C. zeodoria* rhizome flour using MeOH repeated three times. Furthermore, the nonpolar compounds were extracted from the maceration process using n-hexane six times, resulting in a concentrated extract of 43.2 gm (4.32%). To increase its polarity, water was added to the remaining concentrated extract of MeOH and mixed with EtOAc to extract its semipolar compounds. This treatment was repeated three times to obtain 43.2 gm (4.32%) of concentrated extract of EtOAc.

Several reports of previous studies produced diverse extracts. Setyani et al. [10], for instance, obtained 73.68 gm (3.684%) of crude extract in the form of blackish brown gummy from 2 kg of white turmeric rhizome that was extracted by maceration using MeOH for 3 days. The MeOH was immersed for at least 3 days, accompanied by frequent stirring to soften, destroy plant cell walls, and release compounds. In addition, Setyani et al. [10] revealed that from as much as 65 gm of crude extract of white turmeric partitioned respectively using n-hexane, EtOAc, and MeOH, only 30.59 gm (47.06%) of EtOAc, 7.17 gm (11.03%) of n-hexane extract, and 4.31 gm (6.63%) of MeOH were produced. The compounds of *C. zedoaria* rhizome are classified as semipolar compounds.

Another study conducted by Pimrat et al. [32] found that when *C. zedoaria* rhizome was extracted using 95% ethanol and heated by steam at 121ºC for 15 min, it produced a 20.49% concentrated extract with 152 ± 1.8 mg/gm dry curcuminoid. While it was done without steam but heated in an oven at 45ºC, it merely produced 13.37% concentrated extract with 91.95 ± 4.14 mg/gm dry curcuminoid. Marliani et al. [18] discovered that the extract of *C. zedoaria* rhizome extracted using 96% ethanol at 70ºC for 24 h yields a 26.15% ± 5.162% concentrated extract with a total of 84.15 ± 4,142 mg GAE/gm phenolic compound. In comparison, using water (as the solvent) at 70ºC for 24 h produces 26.08% ± 0.813% concentrated extract and a total of 23.463 ± 2.010 mg GAE/gm phenolic.

The difference in the amount of concentrated extract produced is caused by several factors, such as the thickness of the cell walls and cell membranes, the maceration process, and the solvent used. Zhang et al. [20] stated that the extraction result of plants by maceration is mainly determined by the thickness of the cell walls and cell membranes. It occurs because the maceration involves immersing the plants in certain solvents; thus, the pressure inside and outside the cell is different, so that secondary metabolites dissolve from the cytoplasm. The pressure difference could rupture the cell walls and membrane. This is what determines the size of the yield produced in an extraction process done by maceration. The thickness of cell walls is mostly influenced by the genetic factors of the sample. Malahayati et al. [33] suggested that the size of the yield of a concentrated extract is influenced by the maceration process. In this process, not only are secondary metabolites extracted but also all other metabolites. That is why the size of the yield is strongly influenced by the maceration. The type of solvent used also determines the size. Each active compound of each type has its own specifications for dissolving the active compounds.

Afterward, phytochemical screening was done on each concentrated extract to identify the secondary metabolites (Table 1). In the phytochemical screening of each fraction, it was found that the extract of *C. zedoaria* rhizome consists of several essential secondary metabolites such as phenolics, flavonoids, coumarins, and triterpenoids. This screening was used to test the antioxidants and antibacterial bioactivity. MeOH and EtOAc fractions comprise phenolics, flavonoids, and coumarins, which have antioxidant activities [18,33,34]. Whereas the n-hexane fraction comprises triterpenoids, which have a role in antibacterial activity [35,36]. Marliani et al. [18] concluded that when the white turmeric is extracted using 96% ethanol at 70ºC for 24 h, it will produce the highest total phenolic. Ethanol extraction of *C. longa* rhizome yielded 40.80 mg GAE/gm phenolic, as reported in a study by Array et al. [37]. Meanwhile, an extraction using MeOH generates 15.71 mg GAE/gm of phenolic. Lastly, when the rhizome of *C. longa* is extracted using warm water, it produces 7.54 mg GAE/gm phenolic.

### Antioxidant activity test of MeOH, EtOAc, and n-hexane fractions

Once the phytochemical screening was completed, it was continued with the antioxidant activity test by employing the DPPH assay. The antioxidant activity test was carried out on all obtained fractions (MeOH, EtOAc, and n-hexane). It used vitamin C to compare the antioxidant activity because it is a source of natural antioxidants. Tests on all fractions were conducted to qualitatively determine the antioxidant activity of each fraction against free radicals in DPPH by natural compounds of -OH (hydroxy) groups substituted on the benzene ring, such as phenolic, flavonoid, and coumarin. The test was accomplished using a UV-Vis spectrophotometer at a wavelength of 517 mm [28], where the absorbance value obtained after the measurement is converted to an IC_50 _value (inhibitory concentration). IC_50 _is the concentration of compounds that could obtain as much as 50% of the free radicals brought about by DPPH. The efficacy of IC_50 _is determined by its value. The compound’s activity in capturing free radicals from DPPH increases as the IC_50_ value decreases.

The results of the antioxidant activity of each fraction with an IC_50 _value accomplished using spectrophotometry can be seen in Table 2

The IC_50 _value for each fraction of the sample is obtained using a linear regression equation, where the value of the coefficient is substituted with 50 and coefficient *x* is the concentration of extract whose value will be determined through the calculation, as seen in Table 2. By determining the value of *x*, the concentration of extract required to reduce or capture 50% of the free radicals in DPPH can be verified.

The antioxidant activity test was conducted twice, and the absorbance mean was determined. Determining the IC_50_ involves confidently replacing the concentration value with the yield percentage. The average IC_50 _is 153.49 ± 2.66 ppm for the EtOAc fraction and 185.77 ± 3.91 ppm for MeOH. As for the n-hexane fraction, the IC_50 _value is higher (837.92 ± 5.32 ppm). The results imply that EtOAc and MeOH are potent in reducing free radicals generated by DPPH, whereas n-hexane, with a large IC_50 _value, is less powerful in diminishing free radical activities due to the absence of phenolic or flavonoid compounds. Stankovic et al. [34] stated that the lower the IC_50 _value, the more powerful it is in diminishing free radical activities. The higher the IC_50 _value, the less powerful it is at diminishing free radical activities.

**Table 2.** Antioxidant activity of each fraction of *C. zedoaria*

Sample	Concentration	Absorbance	% Inhibition	Regression equation	IC_50_
Vitamin C	50	0.08795	95.715	*y* = 3.8983x + 30.538 *r* = 0.9317	4.53 ± 3.22
	25	0.06255	96.952		
	15	0.11735	94.282		
	5	1.0137	50.609		
Sample	Concentration	Absorbance	% Inhibition	Regression equation	IC_50_
n-hexane	200	1.83845	10.424	*y* = −2.5373x + 0.0627 *r* = 0.9497	837.92 ± 5.32
	100	2.0014	2.485		
	50	2.0230	1.432		
	10	2.2080	−7.581		
EtOAc	200	0.7934	61.343	*y* = 10.153x + 0.2596 *r* = 0.9906	153.49 ± 2.66
	100	1.26615	38.309		
	50	1.60765	21.670		
	10	2.21505	−7.925		
MeOH	200	0.9931	51.613	*y* = 5.6568x + 0.2387 *r* = 0.9375	185.77 ± 3.91
	100	1.3369	34.862		
	50	1.7644	14.032		
	10	2.23265	−8.782		

The antioxidant activity was completed at the laboratory of Natural Chemicals, Chemical Research Centre, National Research, and Innovation Agency, 2022.

The IC_50 _values of EtOAc and MeOH fractions are still in the recommended range to reduce antioxidant activity. Both compounds comprise secondary metabolites that have a crucial role in the process of reducing antioxidant activity. Such metabolites are phenolics, flavonoids, and coumarins containing a hydroxyl (-OH), which functions to stabilize free radicals brought about by DPPH. If the range is between 200 and 1,000 µg/ml, it can be inferred that the fraction or compound is less active in reducing antioxidant activity, though it still has the potential to be an antioxidant [38]. According to Molyneux [39], when a fraction or compound inhibits oxidation with an IC_50_ value below 50 ppm, it unequivocally exhibits powerful antioxidant activity. An IC_50_ value ranging from 51 to 200 ppm indicates moderate antioxidant ability, while values above 200 ppm indicate weak antioxidant activity. In this study, the n-hexane fraction has a quite large IC_50 _value of 837.92 ± 5.32 ppm, which indicates that this fraction does not have secondary metabolites that play a role in inhibiting antioxidant activity [39]. Jun et al. [40] explained that a compound has a strong antioxidant if IC_50 _< 50 mg/l, an active *antioxidant *IC_50_ is 50–100 mg/l, a moderate antioxidant if IC_50_ is 101–250 mg/l, a weak antioxidant if IC_50_ is 250–500 mg/l, and an inactive antioxidant if IC_50 _is > 500 mg/l.

Although Himaja et al. [31] reported strong antioxidant activity in *C. zedoaria* rhizome extract fractionated with ethanol, EtOAc, and water, our study yielded lower results. As much as 100 µg/ml concentrations of ethanol extract, EtOAc, and water showed, respectively, 85.41%, 97.9%, and 98.95% inhibition of DPPH free radicals. Meanwhile, the concentrations of 100 µg/ml petroleum ether and ethanol extracts of *C. zedoaria *rhizome showed 51.04% and 43.75% inhibition of free radicals of DPPH, respectively. The fact that each extract in their study contained bioactive substances is one explanation for this. The extract exhibits high antioxidant activity as a result. Additionally, the crude extract of *C. zedoaria*’s rhizome’s phytochemical screening demonstrates a favorable response to alkaloids, flavonoids, phenolics, terpenoids, and phytosterols [31]. Sumathi et al. [38] confirmed that the extracts of MeOH, ethanol, EtOAc, chloroform, and benzene show considerable antioxidant activity, whereas the extracts of petroleum and water show the least activity.

**Figure 1.**
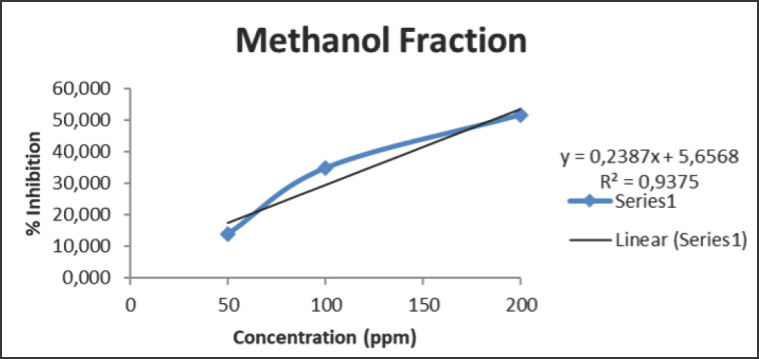
The IC_50_ value of MeOH fraction of *C. zedoaria*

**Figure 2.**
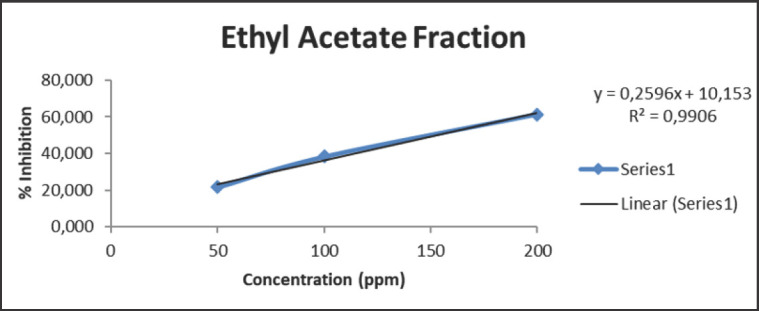
The IC_50_ value of EtOAc fraction of *C. zedoaria*

**Figure 3.**
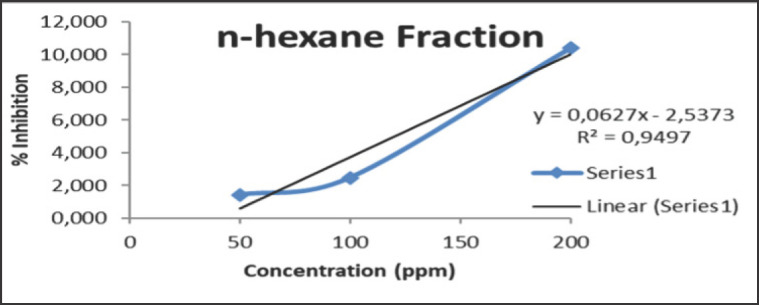
The IC_50_ value of n-hexane fraction of *C. zedoaria*

The IC_50 _value obtained after testing the antioxidant activity of the rhizome extract of *C. zedoaria *in this study was not so powerful as it was carried out on fractions that do not contain pure flavonoid or phenolic compounds. In addition, it is suspected that there are groups of flavonoids and phenolics still bound to the glycosides [41]. The strength of the antioxidant activity of each fraction can be seen in Figures 1–3

From the figures above, it can be seen that the regression equation is obtained by calibrating the relationship between the concentration and maximum absorption on each extract of *C. zedoaria *fraction, which then results in a coefficient *R*^2^ of 0.9906 for the largest EtOAc fraction. The concentration of the extract has a significant impact on the inhibition of antioxidant activity, accounting for 99.06%, while the remaining percentage is attributed to factors like light and temperature [42]. Meanwhile, the ability of the MeOH and n-hexane fractions to reduce the antioxidant activity of DPPH decreases. It can be recognized from the *R*^2^ value of each fraction after calculating the regression equation, which is 0.9375 and 0.9497 for the MeOH and n-hexane fractions, respectively.

The n-hexane fraction has less antioxidant power because it doesn’t have secondary metabolites like phenolics, flavonoids, and coumarins. These metabolites play a crucial role in the process of reducing antioxidant activity. Meanwhile, a decrease occurred in the fraction of MeOH due to the phenolic, flavonoid, and coumarin compounds solving into the EtOAc fraction, which is influenced by the nature of the secondary metabolite compound itself. EtOAc is a semipolar solvent, it may draw both polar and nonpolar substances to curcuminoid, giving it the best antioxidant activity with the lowest IC_50_ value [33]. It is a fact: the antioxidant activity in the rhizome of *C. zedoaria* is directly proportional to a lower IC50 value.

In addition, the polarity level of EtOAc is lower than that of ethanol [43], so semipolar secondary metabolite compounds such as flavonoid and tannin could dissolve in EtOAc. Consequently, less polar plant cell walls are more easily broken down, and phenolic compounds will likewise leave plant cells more easily [44]. Secondary metabolites, which are members of phenolic compounds in plants, have biological activity as antioxidants [45]. Antioxidant activity increases with higher phenolic quantities [46]. Phenolics indeed have antioxidant activity, but the antioxidant activity is not always associated with their total phenolic contents [47].

### Antibacterial activity test by disc diffusion method

The antibacterial test on each extract of *C. zedoaria *fraction was conducted using six types of bacteria namely *E. coli*, *P. aeruginosa*, *S. typhi*, *B. subtilis*, *S. pneumoniae*, and *S. aureus,* and using a positive control of 200 ppm tetracycline, and a negative control of MeOH. The disc method was used to test the antibacterial activity. To determine the activity of each fraction in inhibiting the growth of bacteria, the diameter of the inhibition zone around the disc (which has been treated) was calculated [48]. The antibacterial test description for three fractions of *C. zedoaria *can be seen in Table 3

Antibacterial activity tests, using a disc made of filter paper and extracting diffusion from each fraction whose concentration has been varied, have certain effects on inhibiting the growth of bacteria. The diameter of the inhibition value produced after the experiment in this study seems substantial. The types of bacteria used in this research were Gram-positive bacteria such as *B. subtilis*, *S. pneumoniae*, and *S. aureus,* and Gram-negative bacteria such as *E. coli*, *P. aeruginosa*, and *S. typhi*. This study focuses on the effectiveness of *C. zedoaria *extract, which has been simplified into three fractions. Furthermore, the inhibition zone of the disc which has been diffused by each extract against the growth of Gram-negative and Gram-positive bacteria was measured. The inhibition zones of each extract of MeOH, EtOAc, and n-hexane can be seen in Table 3

**Table 3.** Antibacterial activity in each fraction of *C. zedoaria*

Sample	*Eschericia coli*	*Pseudomonas aeruginosa*	*Salmonella typhii*	*Bacillus subtilis*	*Streptococcus pneumoniae*	*Staphylococcus aureus*
MeOH	7.8	7.7	8.9	7.6	8.7	7.7
	7.7	7.8	8.8	8.8	7.8	8.8
Mean	7.75	7.75	8.85	8.2	8.25	8.25
Std Dev	0.05	0.05	0.05	0.6	0.45	0.55
						
EtOAc	7.9	7.7	7.9	7.8	8.9	7.9
	7.8	7.8	7.8	8.8	9.9	7.8
Mean	7.85	7.75	7.85	8.3	9.4	7.85
Std Dev	0.05	0.05	0.05	0.5	0.5	0.05
						
n-hexane	9.9	7.8	8.9	9.8	8.8	8.8
	9.8	8.8	9.8	9.9	8.9	7.8
Mean	9.85	8.3	9.35	9.85	8.85	8.3
Std Dev	0.05	0.5	0.45	0.05	0.05	0.5
						
Control +	14.625	14.645	14.635	14.205	12.625	14.705
Control -	5	5	5	5	5	5

Antibacterial activity test was accomplished at Microbiology Laboratory, Vaccine and Drug Research Centre, National Research and Innovation Agency, 2022.

The research findings suggest that the extract of *C. zedoaria*, which has been divided into three fractions, may serve as a potential natural compound for combating bacterial infections. From Table 3 above, it can be seen that the n-hexane fraction has better power in inhibiting bacterial growth compared with the MeOH and EtOAc fractions. The n-hexane fraction displayed considerable effectiveness in inhibiting the growth of bacteria, including *E. coli*, *S. typhi*, and *B. subtilis*, as indicated by the diameter of the inhibition zone. While all the fractions demonstrated moderate antibacterial activity, n-hexane exhibited a larger inhibition zone diameter than the other two fractions.

Our finding is different from a study conducted by Indriani et al. [49] that the extract of *C. zedoaria- *against *B. cereus*e examined in triplicate at concentrations of 25%, 50%, 75%, and 100% had a strong inhibitory effect with an average diameter of the inhibition zone of 10.10, 12.33, 13.47, and 19.3 mm, respectively. The positive control shows a strong inhibition zone that is more than 20 mm. Likewise, the antibacterial activity of *S. epidermidis *is also potent, with the average inhibition zones being 11.63, 13.23, 16.00, and 20.03 mm, respectively. Indryani et al. [49] conveyed that the average diameter of the inhibition zone is very strong if it is >20 mm. If it ranges between 10 and 20 mm, then it has strong inhibition power. The inhibition is said to have moderate inhibiting potential if the diameter is between 5–10 mm, and weak if it is <5 mm.

de Tejada et al. [50] explain that the cell wall of Gram-positive bacteria comprises a lone layer of hydrophilic peptidoglycan, rendering it susceptible to the infiltration of polar compounds. This makes it easier for antibacterial compounds to penetrate the cell. Gram-positive bacteria lack an outer membrane but possess a considerably thicker peptidoglycan layer in comparison to Gram-negative bacteria. Furthermore, Prakosa et al*. *[51] elucidated the mechanism of the antibacterial activity of secondary metabolites of *C. zedoaria, *such as saponin, alkaloid, and flavonoid. Flavonoids can destroy the permeability properties of the membrane cells of bacteria and can form extracellular compounds and complex proteins that lyse bacterial membranes [51]. In contrast, alkaloids have antibacterial properties because they can interfere with the components of peptidoglycan. As a result, the formation of membranes is disrupted due to the absence of peptidoglycan in the membranes. Meanwhile, saponins could bind to membrane components of bacteria, such as lipopolysaccharides, disrupting the cell wall’s permeability. *Escherichia coli* and other Gram-negative bacteria possess three distinct layers of walls that make them more resilient against antibacterial agents. The three layers include the cytoplasmic membrane, peptidoglycan, and outer membrane. The outer membrane is made up of asymmetrical lipids, with phospholipids and lipopolysaccharides in the inner and outer layers [50]. The lipoprotein layer is a hydrophobic substance that could block hydrophilic antibacterial from entering the cells so that Gram-negative bacteria are more resistant to antibacterials. That is why it is easier for antibacterial to enter *S. aureus *than *E. coli.*

Our study’s findings contradict those of Malahayati et al [33] regarding the extract of *C. domestica Val. *They found that the fraction of EtOAc with a concentration of 2,000 ppm produced the highest inhibition zone (compared to fractionation using n-hexane and ethanol) of 6.59 ± 0.05 mm (30.44% control positive) against *S. aureus *and 6.29 ± 0.05 mm (25.27% control positive) against *E. coli. *It is because the extract of *C. domestica Val *mixed with EtOAc contains the highest phenolic content, thus having the highest antibacterial activity.

This study found that the highest inhibition zone was in fractionation using n-hexane, due to it containing a considerable amount of triterpenoid and having antibacterial activity [35,36]. The growth-inhibiting potential of MeOH and EtOAc fractions is limited due to the absence of triterpenoids in the former and the restricted activity of the latter, which only inhibits *S. pneumoniae*. Dosoky et al. [15] stated that the rhizome of *C. zedoaria *has essential oil, which particularly consists of 80%–85% sesquiterpenoids and 15%–20% monoterpenoids (15%–20%). Triterpenoids possess the remarkable ability to reduce membrane permeability in bacterial cells by eliminating purines, thus obstructing bacterial growth and potentially inducing cell death. Likewise, flavonoids contain phenol that could also destroy the membranes of bacteria cells, especially plasma membranes, which then inhibit bacterial growth, followed by death in the bacteria [49].

**Figure 4.**
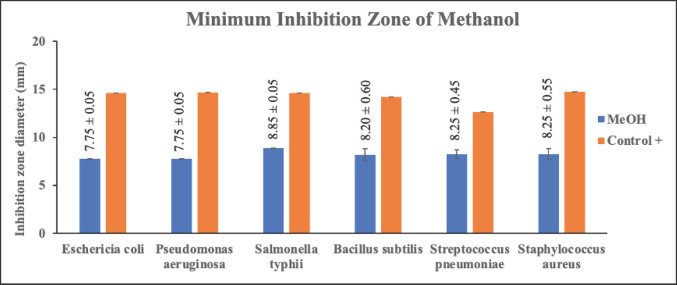
Test results for the antibacterial activity of MeOH fraction of *C. zedoaria*

**Figure 5.**
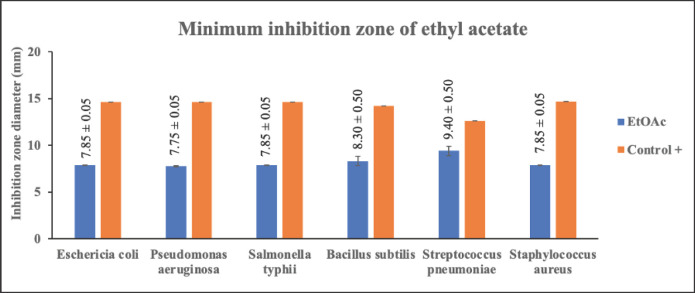
Test results for the antibacterial activity of EtOAc fraction of *C. zedoaria*

**Figure 6.**
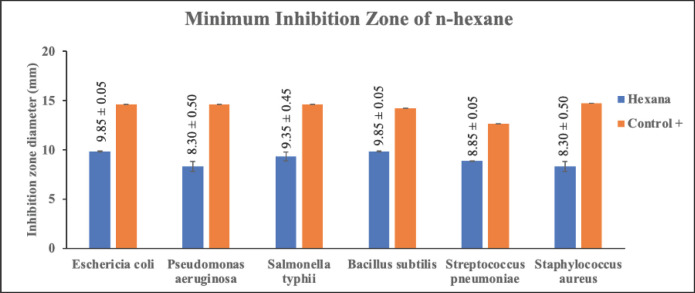
Test results for the antibacterial activity of n-hexane fraction of *C. zedoaria*

Alkaloids have antibacterial properties because they can interfere with the components of peptidoglycan. As a result, the formation of membranes is disrupted due to the absence of peptidoglycan in the membranes. Meanwhile, saponins could bind to the membrane components of bacteria, such as lipopolysaccharides, thereby disrupting the permeability of the cell wall [51]. Bugno et al. [12] reported that the extract of *C. zedoaria *at a dosage of 100, 250, 500, and 1000 mg/ml is effective against such bacteria as *S. mutants, E. faecalis, S. aureus, *and *C. albicans *and could reduce as much as 99.99% of the microbial population. The effectiveness of each fraction of *C. zedoaria *in inhibiting the growth of bacteria (Gram-positive and Gram-negative bacteria) can be seen in Figures 4–6

### MIC assay

For each fraction (MeOH, EtOAc, and n-hexane), the MIC assay was used to determine the lowest concentration of *C. zedoaria* extract needed to inhibit the antibacterial activity of the examined bacteria. The results were obtained after incubation for 24 h. An observation was completed by turbidimetry or visual observation of the turbidity against the solution in a microplate. The results of the observation are presented in Table 4. The results revealed that the n-hexane fraction has an excellent capability for inhibiting bacterial growth, followed by EtOAc and MeOH.

Based on the observation, it is evident that the fraction of n-hexane exhibits the most effective bacterial growth inhibition activity compared to the other two fractions tested. The results of this MIC test are directly proportional to the ones gained by testing the bacterial growth using the disc or observing the clear zone. Based on the MIC assay, it was known that the lowest concentration necessary for an n-hexane fraction to inhibit bacterial growth was 3.125 ppm for *E. coli*, 1.562 ppm for *P. aeruginosa*, 7.810 ppm for *S. typhi*, 4.687 ppm for *B. subtilis*, 6.250 ppm for *S. pneumoniae*, and 7.810 ppm for *S. aureus*. Of the three fractions of *C. zedoaria*, n-hexane has a good inhibitory activity for the growth of *S. aureus* and *S. typhi*. The fractions of EtOAc and MeOH, on the other hand, have a less substantial effect on inhibiting bacterial growth. The difference in the fraction and secondary metabolites within each fraction has a significant influence on the inhibition of bacterial growth. This occurs because the types of secondary metabolites in each fraction are different and have diverse activities [36]. That is why the fractions of EtOAc and MeOH are not truly active in inhibiting bacterial growth.

### MBC assay

The goal of the MBC assay for each *C. zedoaria* extract is to assess the inhibitory activity of each fraction (MeOH, EtOAc, and n-hexane) against six types of bacteria., namely *E. coli*, *P. aeruginosa*, *S. typhi*, *B. subtilis*, *S. pneumoniae, *and *S. aureus*. The observation in this study was done after 24 h of incubation by calculating the effectiveness of each extract at various concentrations of 2,500, 5,000, 7,500, and 10,000 ppm. The concentration of MBC was evaluated by observing the bacterial growth on the media that had been incubated. The bacteria were plated in the media according to the lowest concentration that inhibits bacterial growth, and the concentration can be recognized from the MIC obtained. The minimum concentration needed for each extract of *C. zeodoria* to kill the tested bacteria can be seen in Table 5

After conducting tests, it was found that n-hexane proved to be the most effective in eliminating bacteria, requiring only a concentration of 2,500 ppm for all strains. In contrast, MeOH and EtOAc fractions necessitated a higher concentration, ranging from 2,500 to 7,500 ppm, to achieve the same outcome. Our study aligns with previous research on rose apple leaves, showing that the n-hexane fraction is effective in inhibiting the growth of both *S. aureus* and *E. coli *bacteria [52]. This MBC assay shows that the results correlate to the phytochemical screening test done using the disc. The results provide further information that n-hexane from the extract of *C. zedoary* has an active role in inhibiting as well as killing the bacteria used in this experiment. While the EtOAc and MeOH fractions require further antibacterial activity testing as they still inhibit the growth and kill the bacteria.

**Table 4.** The comparison of values of MIC activity of each fraction against test bacteria.

	*Eschericia coli* (ppm)	*Pseudomonas aeruginosa* (ppm)	*Salmonella typhi* (ppm)	*Bacillus subtilis* (ppm)	*Streptococcus pneumoniae* (ppm)	*Staphylococcus aureus* (ppm)
**Fraction of MeOH**	**6.250**	**6.250**	**3.125**	**12.500**	**12.500**	**3.125**
**Fraction of EtOAc**	**4.687**	**4.687**	**3.125**	**9.375**	**9.375**	**3.125**
**Fraction of n-hexane**	**3.125**	**1.562**	**7.810**	**4.687**	**6.250**	**7.810**
**Control +**	**7.810**	**7.810**	**7.810**	**7.810**	**7.810**	**7.810**
**Control -**	**100.000**	**100.000**	**100.000**	**100.000**	**100.000**	**100.000**
	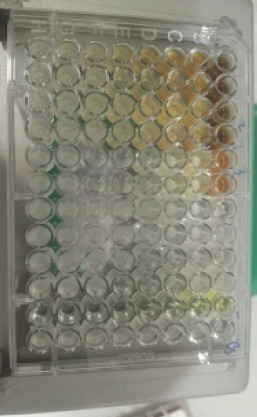	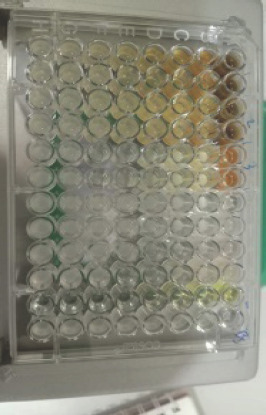	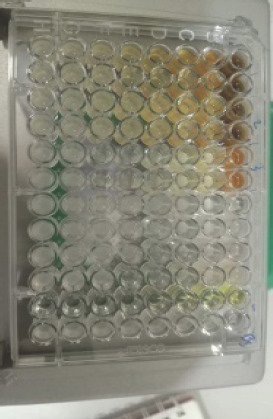	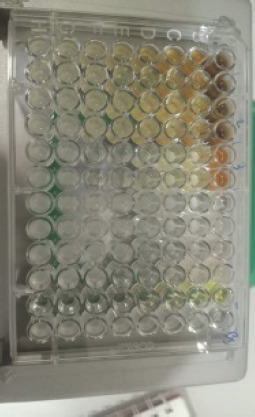	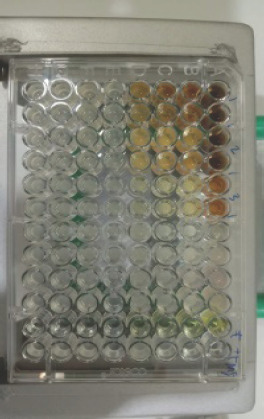	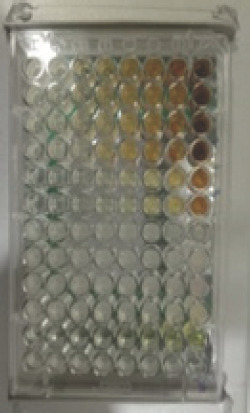

MIC test was accomplished at Microbiology Laboratory, Vaccine and Drug Research Centre, National Research, and Innovation Agency, 2022.

**Table 5.** The comparison of the value of MBC activity from each fraction of *C. zedoaria*

MBC from each fraction of *C. zedoaria*						
Sample	Bacteria/Concentration					
	*Escherichia coli*	*Pseudomonas aeruginosa*	*Salmonella typhi*	*Bacillus * *subtilis*	*Streptococcus pneumoniae*	*Staphylococcus aureus*
MeOH fraction	>5,000	>5,000	>2,500	>7,500	>7,500	>2,500
EtOAc fraction	>2,500	>5,000	>2,500	>5,000	>5,000	>2,500
n-hexane fraction	>2,500	>2,500	>2,500	>2,500	>2,500	>2,500
Tetracycline	>50	>50	>50	>50	>50	>50
MeOH	>100,000	>100,000	>100,000	>100,000	>100,000	>10,0000

MBC test was accomplished at Microbiology Laboratory, Vaccine, and Drug Research Centre, National Research, and Innovation Agency.

The weaknesses of our study are as follows: 1. We have not been able to determine the pure compound that we can purify from the active fraction in terms of antioxidant and antibacterial activity testing. 2. To find out the active compounds, we have to isolate and purify them as well as characterize the secondary metabolic compounds that we have succeeded in purifying, so we have not tested the antioxidant and anticancer activities of these pure compounds. 3. This research is at an early stage in determining the active fraction of turmeric for antioxidant and antibacterial. Further research on the use of this white turmeric as an antioxidant and antibacterial is urgently needed.

## Conclusion

Based on the research outcome explained above, it can be concluded that the extract of *C. zedoaria* fractionated by using MeOH produces alkaloids, phenolics, flavonoids, saponins, and coumarins. In addition to alkaloids, phenolics, flavonoids, saponins, and coumarins, the fractionation using EtOAc also produces triterpenoids. Whereas the one accomplished using n-hexane merely yields alkaloids and triterpenoids. Furthermore, EtOAc and MeOH fractions have good activity in reducing free radicals generated by DPPH, with an average IC_50 _value of 153.49 ± 2.66 and 185.77 ± 3.91 ppm, respectively. However, n-hexane has weak antioxidant activity, with an average IC_50 _value of 837.92 ± 5.32 ppm. All fractions have moderate antibacterial activity, but the extract of n-hexane from *C. zedoary* has better antibacterial activity compared to MeOH and EtOAc. The lowest concentration required is 2,500 ppm for all types of bacteria.

## List of abbreviations

CRD, Chronic Respiratory Disease; DPPH, 2,2-Diphenyl-1-picrylhydrazyl; EtOAc, ethyl acetate; IC_50_, Inhibitory Concentration; nm, nanometer; MeOH, Methanol; MBC, Minimum Bactericidal Concentration; MIC, Minimum Inhibitory Concentration; mm, millimeter; μl, microliter; ml; R^2^, Correlation coefficient value; ppm, part per Million.
